# Detecting Progression of Treated Choroidal Melanomas: Is Ultrasonography Necessary?

**DOI:** 10.3390/cancers13225832

**Published:** 2021-11-20

**Authors:** Guy S. Negretti, Umiya Harley, Amit K. Arora, Gordon Hay, Mandeep S. Sagoo, Bertil E. Damato

**Affiliations:** 1 Ocular Oncology Service, Moorfields Eye Hospital, London EC1V 2PD, UK; umiya.harley@nhs.net (U.H.); amit.arora@nhs.net (A.K.A.); gordon.hay3@nhs.net (G.H.); mandeep.sagoo1@nhs.net (M.S.S.); bertil.damato1@nhs.net (B.E.D.); 2 Ocular Oncology Service, Royal Hallamshire Hospital, Sheffield S10 2JF, UK; 3 Department of Ophthalmology, St. Bartholomew’s Hospital, London EC1A 7BE, UK; 4 UCL Institute of Ophthalmology, 11-43 Bath Street, London EC1V 9EL, UK; 5 NIHR Biomedical Research Centre, at Moorfields Eye Hospital and University College London Institute of Ophthalmology, 11-43 Bath Street, London EC1V 9EL, UK; 6 Nuffield Laboratory of Ophthalmology, Department of Clinical Neurosciences, University of Oxford, John Radcliffe Hospital, Oxford OX3 9DU, UK

**Keywords:** uveal melanoma, recurrence, long-term surveillance, imaging modalities

## Abstract

**Simple Summary:**

Long-term surveillance following radiotherapy for choroidal melanoma is important for detecting recurrence. There is a perceived notion that regular ultrasonography is required to detect recurrence. The skills required to perform ocular ultrasound are not widely available, which can prevent patients being seen close to home. We aimed to determine whether local treatment failure can reliably be detected with colour fundus photography alone. We found that in 74 out of 75 patients (98.7%), with a clear view of their fundus, recurrence could be detected using colour photography alone. One patient with a clear fundal view developed extraocular extension which was detected on ultrasound without visible change in the intraocular part of the tumour. We conclude that most treated choroidal melanomas can be monitored without ultrasonography if they can be adequately imaged with colour photography.

**Abstract:**

Prompt detection and treatment of local treatment failure after radiotherapy for choroidal melanoma optimises any opportunities for conserving vision and the eye, possibly reducing an increased risk of metastatic disease. Long-term surveillance is therefore required but is hampered by the perceived need to perform ultrasonography, which may not be available at a patient’s local hospital. The aim of this study was to determine whether local treatment failure can reliably be detected with colour fundus photography alone, and, if so, in which patients. Patients were included in the study if diagnosed with local treatment failure between April 2016 and February 2021 after eye-conserving therapy for choroidal melanoma. Wide-field colour and fundal autofluorescence (FAF) images, optical coherence tomography (OCT), and ultrasonography (US) were analysed by two of the authors (GN and UH). The cohort included 87 patients with local treatment failure. In 75 patients with clear media, tumour progression was detected by colour photography alone in 74 (98.7%) patients. Sensitivity was not increased by the addition of either OCT or AF. One patient with clear media developed extraocular extension detected with US without visible change in the intraocular part of the tumour. In the other 12 patients, US was required because of opaque media and a consequently poor fundal view. Local treatment failure after radiotherapy for choroidal melanoma is detected in 98.7% of cases with colour photography when the media are clear. Ultrasonography is useful when photography is prevented by opaque media or tumours having locations in the far periphery.

## 1. Introduction

In patients with choroidal melanoma, five-year local treatment failure is reported in approximately 2–10% of patients after brachytherapy, 3.5% of patients after proton beam radiotherapy, 19% after transpupillary thermotherapy (TTT) and 62% after photodynamic therapy (PDT) [1,2,3,4,5]. Early detection and treatment of tumour progression improves the chances of conserving vision and the eye, perhaps also preventing metastatic spread in some cases [6]. Local tumour recurrence after radiotherapy is associated with increased mortality, although it is not known whether the recurrence is the cause of metastatic disease or merely an indicator of increased tumour aggressivity [7].

Previously, we monitored all treated choroidal melanomas at our hospital, performing ultrasonography (US) at every visit. Because of the COVID-19 pandemic, many patients were reluctant to travel to our centre and were therefore discharged to their local hospital for long-term surveillance. Several local ophthalmologists expressed concern about taking over the care of these patients because they did not have the equipment or the skills needed to perform accurate ocular ultrasonography.

We therefore performed this study to determine which treated choroidal melanomas, if any, can safely be monitored without ultrasonography.

## 2. Materials and Methods

This is a single-centre case series study. The electronic patient records were reviewed to identify all patients who were diagnosed with local treatment failure between April 2016 and February 2021 after eye-conserving therapy at our centre. All the patients in the study had been discussed at a multidisciplinary team (MDT) meeting where at least two senior ocular oncologists had agreed on the diagnosis of treatment failure.

All patients at Moorfields are examined at every follow-up visit by ophthalmoscopy, wide-field colour and fundus autofluorescence (FAF) imaging (Optos California (Optos plc, Dunfermline, Scotland)), spectral-domain optical coherence tomography scan (OCT) (Heidelberg Spectralis (Heidelberg Engineering GmbH, Heidelberg, Germany)) and B-scan ultrasonography using a 15L8 transducer probe (Acuson Sequoia (Siemens, Healthineers, Frimley, Camberly, UK)).

The detection of local treatment failure was categorised as being achieved by: (A) photography, if there was a visible increase in basal tumor dimensions and/or if a convincing increase in height was noted; (B) OCT, if increased subretinal fluid was detected or if an increase in thickness could be appreciated with this modality; (C) fundus autofluorescence imaging, if hyperfluorescent dusting of lipofuscin appeared or if margins of the tumour convincingly increased; and (D) ultrasonography, if the measured tumor thickness increased by more than 0.5 mm. Due to the limits of ultrasound resolution, for a recurrence to be diagnosed on the basis of ultrasound alone, an increase in tumour thickness greater than 0.5 mm or a diameter exceeding 2 mm had to be observed on more than one occasion.

In this study, after undergoing a pilot study and ensuring agreement between them about what did and did not constitute a recurrence on imaging, two of the authors (GN and UH, both of whom have sub-specialty training in ocular oncology) retrospectively analysed the imaging of half the study participants each in order to determine which modalities provided diagnostic evidence of recurrence. If there was any question about whether a recurrence was present on imaging or not, it was marked as negative. The authors thus only identified obvious recurrences and marked any that were subtle or questionable as negative. If a patient had undergone multiple procedures to treat more than one recurrence, then the most recent recurrence was used for the purpose of this study. Marginal tumour extension was identified by assessing distances between tumour margins and nearby retinal landmarks (e.g., blood vessels). Where marginal extension was identified, the area of largest extension was measured. There was no defined size for what constituted marginal tumour extension and what did not. It had to be convincing to the researcher analysing the image. If there was any question about whether there was marginal extension or not, it was marked as negative for tumour extension.

Descriptive statistics were used to estimate mean ± standard deviation (SD) (range) when normally distributed, and median (interquartile range [IQR]) when the data were skewed. This study was approved by the Institutional Review Board at Moorfields Eye Hospital (CA20/ONC/796) and adhered to the tenets of the Declaration of Helsinki.

## 3. Results

Between 1 April 2016 and 1 February 2021, 87 patients were diagnosed with recurrent choroidal melanoma at Moorfields Eye Hospital. This included 49 (56%) males and 38 (44%) females, with the tumour located in the right and left eye in 42 (48%) and 45 (52%) cases respectively. The mean age of patients at diagnosis of the most recent recurrence was 66.0 years (SD, 12.5). The median time between initial treatment and the most recent recurrence was 2.9 years (IQR 6.05).

The median largest basal diameter and thickness of the tumours on ultrasonography at the time of initial diagnosis were 9.4 mm (IQR 4.1) and 3 mm (IQR 2.2), respectively. The median tumour distances to the fovea and optic disc margin were 2.5 mm (IQR 5.1) and 2.6 mm (IQR 4.6), respectively. Forty-eight (55%) tumours extended within 2 disc-diameters of the optic disc margin, with 22 (25%) involving the optic disc. The initial treatment modalities employed are shown in Table 1.

The clinical features of tumour recurrences are shown in Table 2.

**Figure 1:**
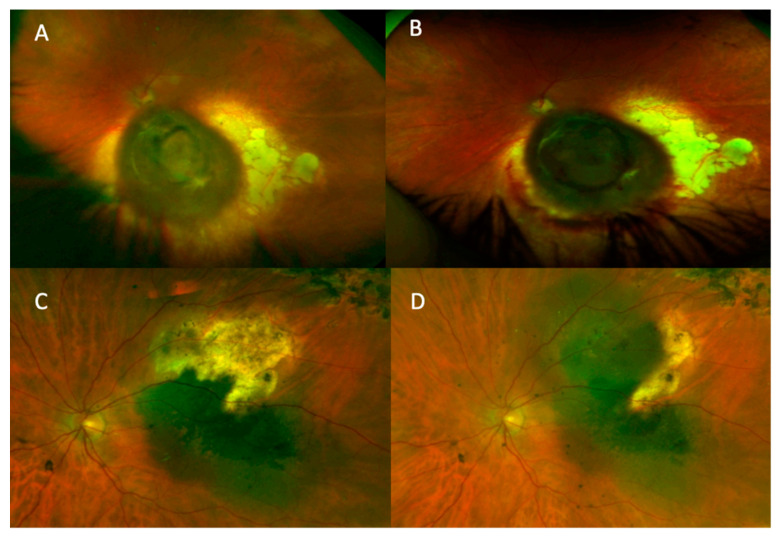
Tumour recurrences as seen in wide-field colour photographs. (**A**,**B**) demonstrate how increased thickening of the tumour can be observed with colour photography. (**C**,**D**) demonstrate lateral tumour extension.

In the 75 eyes with clear media, 74 (98.7%) of the recurrences could be diagnosed on the basis of wide-field photography or anterior segment photography alone, as illustrated in Figure 1. Slitlamp examination and anterior segment photography identified 2/75 (2.67%) patients who had anterior extrascleral extension. This gave a true positive rate for photography in the detection of recurrence, in those with clear media, of 98.7%, and a false negative rate of 1.3% (there were no true negatives or false positives because all cases included in this study were recurrences).

**Figure 2:**
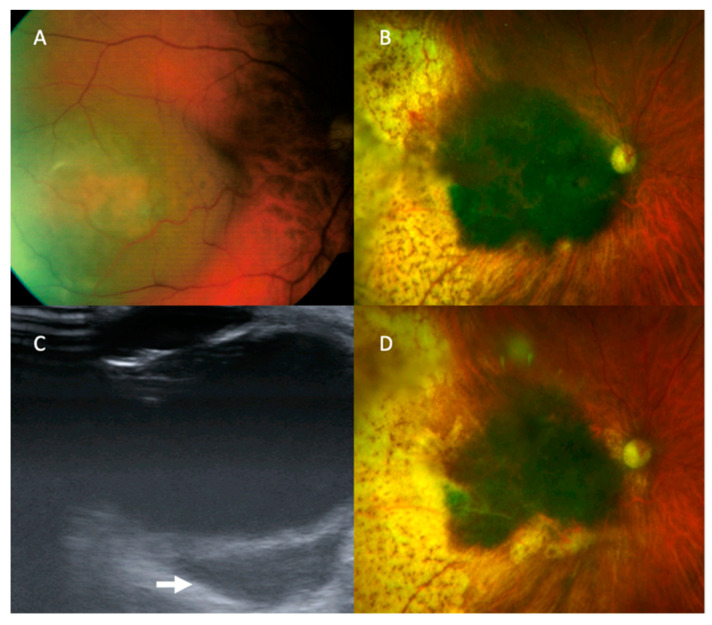
Tumour recurrence in the one patient with clear media whose recurrence could not be diagnosed on colour photography. (**A**) The tumour at presentation. (**B**) The tumour following initial treatment with plaque brachytherapy and salvage therapy with proton beam radiotherapy. (**C**) B-scan ultrasound demonstrating extrascleral extension (arrow points to area of extrascleral extension). (**D**) Colour photograph of the tumour at the time extrascleral extension was diagnosed.

There was one recurrent tumour that was detected by US alone. The patient was initially treated with plaque brachytherapy at the age of 65 years for a macular choroidal melanoma in the right eye (Figure 2). The tumour had a basal diameter of 8 mm, a thickness of 4 mm, and its posterior margin was 3 mm from the optic disc edge. Five months later, the tumour showed marginal growth towards the optic disc and increased thickness, which was treated with proton beam radiotherapy. 97 months after the initial treatment (and 86 months after the salvage therapy), US revealed an extraocular tumour, measuring 15 mm × 7.6 mm × 17.1 mm in size and located temporally adjacent to the optic nerve. Colour photography and other imaging showed no change. The patient underwent enucleation which demonstrated choroidal malignant melanoma of predominantly epithelioid-cell type with brisk intratumoural lymphocytic infiltrate with extrascleral extension involving the surgical resection margins (AJCC grade pT4e). Staging CT prior to the patient’s enucleation demonstrated widespread metastatic disease and at their latest follow up, 10 years following the initial diagnosis, he was being treated with Nivolumab immunotherapy.

Of the 75 tumours in eyes with clear media, the entire tumour margin could be imaged with colour photography in 65 cases (87%). In the 48 cases with marginal tumour recurrence and adequate image quality to measure, the largest marginal extension measured <1 DD in eight patients, 1–2 DD in 25 and >2 DD in 15.

At the time of detection of local treatment failure, 12 (13.8%) tumours could not be visualised by ophthalmoscopy or colour photography because of cataract (7), vitreous haemorrhage (4) or hyphaema (1). For these 12 tumours with opaque media, the diagnosis of treatment failure was based on ultrasound-measured increased tumour thickness, greater basal diameter, and both, in eight, two and two cases, respectively. The increase in tumour thickness had a median of 2 mm (range, 1.5–5.4) and the increased basal diameter had a median of 6.2 mm (range, 2.4–11.2).

Neither OCT nor FAF revealed any recurrence that was missed with colour photography. OCT simply supported the diagnosis of recurrence already made with colour photography in 44/74 (59.5%) patients. Recurrence was demonstrated with OCT either by increased subretinal fluid (11/44, 25%), increased thickness (31/44, 70.5%) or both (2/44, 4.5%). Wide-field FAF supported the diagnosis of recurrence already made with colour photography in 49/74 (66%) patients. Recurrence was diagnosed either by observing a border change to the tumour on the autofluorescence image (31/49, 63%), by increased hyperfluorescent lipofuscin in an area of recurrence (6/49, 12%) or by observing both a border change and increased lipofuscin (12/49, 24%).

**Figure 3:**
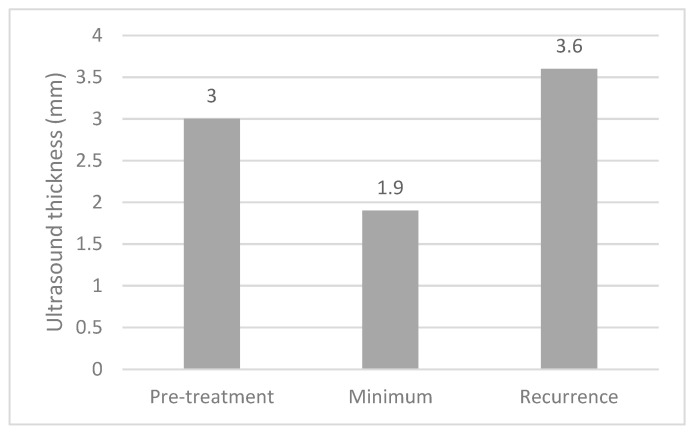
Bar chart demonstrating the median thickness of choroidal melanomas at diagnosis, at their minimum post-treatment and at recurrence (all measurements in mm). Paired *t*-tests demonstrated a significant difference between pre-treatment and minimum measurements and between minimum measurements and recurrence (*p* < 0.001).

The median thicknesses of the melanomas on ultrasound at diagnosis, at their minimum post treatment and at recurrence are shown in Figure 3. Paired *t*-tests demonstrated significant differences between pre-treatment and minimum measurements (*p* < 0.001) and between minimum measurements and recurrence (*p* < 0.001).

## 4. Discussion

### 4.1. Main Findings

The most important finding of this study is that in eyes with clear media, 98.7% of local treatment failures were detected by colour photography alone. In eyes with clear media, the only recurrence that was detected by ultrasonography alone was located extraocularly. Recurrences diagnosed with colour photography also showed evidence of progression with OCT and FAF in 59% and 66% of patients, respectively.

### 4.2. Strengths and Weaknesses of Study

Recurrences following primary treatment for uveal melanoma are rare, with previously published five-year recurrence rates following plaque brachytherapy and proton beam radiotherapy between 2% and 10% [1,2,3]. The strengths of this study are the large cohort size and the use of multiple imaging techniques (wide-field colour photography, autofluorescence imaging, spectral domain OCT and ultrasound).

A weakness of this study is that all the images were analysed by two ocular oncologists trained in identifying subtle recurrences.

### 4.3. Comparison with Other Studies

Our results complement a recent study by Roelofs et al., which demonstrated the utility of colour fundus imaging alone for monitoring the progression of untreated choroidal melanocytic tumours [8]. In their study, they demonstrate a 97% sensitivity for detecting progression of choroidal melanocytic tumours with colour fundus photography and without US.

### 4.4. Clinical Implications

Traditionally, ocular oncology patients have tended to undergo all their follow-up assessments at an ocular oncology centre. Surveillance closer to their home would reduce travel expenses, time off work or away from home and stress caused by fear of COVID-19 infection. There is a significant desire amongst patients and practitioners for ocular monitoring of treated choroidal melanomas to be performed closer to the patient’s home and, if necessary, having images of the tumour analysed by experts working remotely [9,10,11]. Recent technological advances have made ophthalmic imaging more accessible to healthcare workers in optometry and smaller ophthalmology practices; however, the lack of sensitive ocular ultrasonography in these units has prevented them from monitoring treated choroidal melanomas. The present study is particularly significant because it demonstrates that ultrasound may not be necessary when the treated tumour can be monitored by sequential colour photography. Avoidance of ultrasonography should prevent false positive diagnosis of recurrence because of inaccurate measurement of tumour dimensions (e.g., including the sclera and/or retina in the thickness measurement). 

As mentioned above, OCT and FAF did not reveal any recurrences that were not detected with colour photography. This study therefore suggests that they are not essential, although they may be useful in providing supportive evidence of tumour progression in some cases.

The one patient with clear media whose recurrence was detected with US alone was exceptional, because the tumour was extraocular and because it had developed after salvage treatment for a previous recurrence with proton beam radiotherapy, which normally has a high success rate. Nevertheless, this raises the question of which tumours require US for adequate monitoring. We tentatively suggest that US is indicated for: patients with opaque media and peripheral tumours that cannot be adequately assessed by ophthalmoscopy and/or colour photography.

### 4.5. Research Implications

There is a need for further studies to determine how well ophthalmologists and optometrists can identify tumour recurrence with colour photography or, if they have no access to wide-field imaging, with ophthalmoscopy alone.

There is scope for evaluating the clinical impact of tumour recurrences detected by non-oncologists. A better understanding of how recurrence size and location influence ocular outcomes would help identify patients who can safely be monitored by non-oncologists.

## 5. Conclusions

In conclusion, most treated choroidal melanomas can be monitored without ultrasonography if they can be adequately imaged with colour photography. Further studies are indicated to audit surveillance by non-oncologists and to define more precisely the indications for ultrasonography.

## Figures and Tables

**Table 1:** Tumour characteristics at diagnosis and the treatment modalities employed for their initial treatment.

Study Eye Characteristics	Number (%), *N* = 87
**Laterality**	
Right	42 (48%)
Left	45 (52%)
**Sex**	
Male	49 (56%)
Female	38 (44%)
**Tumour quadrant**	
Inferotemporal	31 (36%)
Superotemporal	29 (33%)
Superonasal	17 (20%)
Inferonasal	10 (11%)
**Primary Treatment Modality**	
Plaque brachytherapy	61 (70.1%)
Proton beam radiotherapy	9 (10.3%)
PDT	14 (16%)
TTT	2 (2.3%)
Surgical resection	1 (1.2%)

**Table 2:** Clinical features of recurrences together with the treatment modalities employed for their treatment.

Recurrence Characteristics	Number (%), *N* = 87
**Clinical features of recurrence**	
Central	29 (33.3%)
Marginal	22 (25.3%)
Central and Marginal	29 (33.3%)
Extraocular extension	3 (3.4%)
Extraocular and marginal extension	4 (4.6%)
**Recurrence treatment modality**	
Plaque brachytherapy	15 (17%)
Proton beam radiotherapy	32 (37%)
PDT	1 (1%)
TTT	5 (6%)
Enucleation	34 (39%)

## Data Availability

The data presented in this study are available on request from the corresponding author.
